# Editorial: Immunosenescence in organ transplantation

**DOI:** 10.3389/frtra.2024.1422358

**Published:** 2024-06-14

**Authors:** Jasper Iske, Hao Zhou

**Affiliations:** ^1^ Department of Cardiothoracic and Vascular Surgery, Deutsches Herzzentrum der Charité (DHZC), Berlin, Germany; ^2^ Charité-Universitätsmedizin Berlin, Corporate Member of Freie Universität Berlin and Humboldt-Universität zu Berlin, Berlin, Germany; ^3^ German Center for Cardiovascular Research (DZHK), Partner Site Berlin, Berlin, Germany; ^4^ Berlin Institutes of Health at Charité – Universitätsmedizin Berlin, Berlin, Germany; ^5^ Division of Transplant Surgery, Department of Surgery, Brigham and Women’s Hospital, Harvard Medical School, Boston, MA, United States

**Keywords:** senescence, senolytic, Senescence-associated Secretory Phenotype (SASP), transplantation, alloimmunity, aging, senescent cell, solid organ transplantation

**Editorial on the Research Topic** Immunosenescence in organ transplantation

## Introduction

Aging is a complex biological phenomenon that manifests at the cellular, tissue, and organismal levels. It is increasingly clear that rich dynamics underlie biological aging, yet the fundamental nature of this process remains poorly understood. Specifically, inter-tissue dynamics of biological age, and how this may change over the lifespan, have received little attention.

Organ transplantation represents an exceptional, clinically relevant example, with donor/recipient age-discrepant combinations being a clinical routine to meet the ever-increasing demands. Age limits in organ transplantation have been raised drastically for both donors and recipients. Increasing amounts of organs are being utilized to meet the ever-growing demand, and older patients have shown the greatest proportional increase as transplant recipients over the last decade. With patients desperately awaiting a life-saving organ, these procedures are frequently performed heterochronic, i.e., with disparate chronological ages of the donor. Notably, increasing donor age, independent of recipient age, has been correlated with a higher rate of acute allograft rejection (1, 2).

Cellular senescence constitutes a molecular underpinning of aging characterized by irreversible growth arrest and cellular dysfunction. A cardinal feature of senescent cells constitutes a distinct, pro-inflammatory secretome consisting of cytokines (IL-6, IL-8, TNF-α), chemokines (CCL2, CCL20) and matrix remodeling enzymes termed the “Senescence-associated Secretory Phenotype” (SASP). The SASP has been delineated to promote age-related tissue dysfunction, age-associated chronic diseases and organismal aging, impairing tissue homeostasis and impeding neighboring cell function. In recent years, the accumulation of senescent cells in old donor organs has been characterized as crucial driver of ischemia-reperfusion injury and allo-immune responses, while also impairing recipient outcomes and altering the efficacy of immunosuppressive regimens.

## The significance of cellular senescence in organ transplantation

The accumulation of senescent cells has been identified as a critical driver for promoting the immunogenicity of older organs. Mechanistically, ischemia reperfusion injury leads to a systemic release of cell-free mitochondrial DNA (cf-mt-DNA) deriving from senescent cells, which in turn triggers IL-17-dominated alloimmune responses in transplant recipients (3). Hereby, dendritic cells were found as crucial drivers communicating augmented immunogenicity by promoting CD4^+^ T cells derived IL-17 production leading to compromised survival of older donor organs (4). Moreover, senescence of intragraft T cells has been characterized as an instigator of age-specific organ dysfunction through augmented SASP-related signaling accelerating organ injury and potentially contributing to the limited lifetime of organ transplants (5–9).

More recent studies furthermore deciphered mechanisms by which the transplantation of older organs induces senescence in younger recipients, promoting age-related pathologies (10). Notably, 30 days after transplantation of an older donor organ, augmented frequencies of senescent cells in various recipient organs including draining lymph nodes, livers and hindlimb muscles could be observed associated with a systemic increase of SASP factors including mt-DNA. These findings went along with compromised physical performance and impaired spatial learning and memory abilities which may be of translational relevance not only for future age-mismatched organ transplantations but also for those that have already been performed (11).

Since transplantation of an allogeneic donor organ exposes the recipient immune system to repetitive antigen stimulation, it may not only promote somatic cell senescence but also accelerate immunosenescence (12). Indeed, various studies have demonstrated augmented numbers of senescent T cells linked to compromised telomere lengths following kidney transplantation (13, 14). Of translational relevance, higher frequencies of both, senescent CD4^+^ and CD8^+^ T cells correlated tightly to infections (13) and exhibited inflammatory profiles (14).

In clinical transplantation, T cell immunosenescence has been the main driver of less frequent acute rejections in older transplant recipients. However, accelerated T cell immunosenescence has also been linked to an altered responsiveness to immunosuppressive therapy. Thus, enhanced immunosuppressive effects have been observed for tacrolimus (15) and rapamycin (16) while CTLA4-Ig appeared to be less effective in aged recipients (17).

In clinical practice, older and younger transplant patients are usually treated with the same immunosuppressants and comparable drug levels (18) while strategies of “just” lowering dosages of established immunosuppressants in older patients have not always been successful (19, 20).

Recent evidence has furthermore suggested that age-associated hormone changes in recipients may affect allo-immune responses and transplant outcomes, highlighting the importance of considering age- and sex-specific factors in transplant medicine (21).

## Future perspectives

A promising therapeutic approach targeting the augmented immunogenicity of older donor organs while impeding transplantation-derived senescence induction, lies in the application of drugs that selectively clear senescent cells, known as senolytics (22). Noteworthy, senolytics are already undergoing several clinical trials with encouraging outcomes. Consequently, transplantation emerges as a captivating field for further exploration and application. In experimental models, treating old donor animals with senolytics, prior to transplantation, cleared senescent cells and reduced cf-mt-DNA release, thereby attenuating immune responses and improving the survival of old cardiac allografts, comparable to young donor organs (3). Moreover, senolytic treatment improved physical function of young recipients receiving old donor organs (11).

Although therapeutic benefits of senolytics have accelerated their journey from discovery to clinical trials, cell type specific efficacy, bioavailability and the off-target side effects require further investigation particularly with a view on long-term outcomes. Novel senolytics drugs with higher specificity, selectivity and even greater efficacy are currently under investigation while research for novel compounds is ongoing (23).

For their application in transplantation, the most efficacious deployment of senolytics needs to be evaluated. Hereby, treatment of the donor, the recipient, and the organ itself by recruiting *ex vivo* perfusion systems (24, 25) need to be compared to ensure most efficient treatment while sparing side effects.

To date, transplantation studies have mainly focused on the graft's effect on the recipient's environment. However, studying how the recipient's environment modulates the graft may uncover further mechanisms regulating long term graft outcomes and chronic rejection. Since the transplantation of an old donor organ into a young recipient was sufficient to accelerate aging processes, transplanting a young donor organ into an old recipient is likely to induce senescence in the donor organ (10).

In contrast, the effect of young recipient age on old graft function, may have beneficial effects. Supporting this concept, studies on age-mismatched muscle transplantation in mouse models revealed that the regenerative capacity of skeletal muscle grafts aligns with the age of the recipient (26). Muscle grafts from elderly donors namely exhibited enhanced regenerative abilities when transplanted into young recipients (26). Furthermore, heterochronic parabiosis (HPB) models have provided evidence for rejuvenating aged organ function through the infusion of young blood into older organisms affecting a broad range of organs (27, 28), while the actual identities of rejuvenating factors remain a matter of debate and need further exploration.

## Conclusion

Collectively, the accumulation of senescent cells poses a risk towards utilizing older organs as an untapped source for expanding the donor pool. Utilizing senolytics may provide a novel approach to face this challenge but is currently in the initial phase of clinical realization. Investigating molecular patterns underlying age-mismatched transplantation may furthermore reveal novel mechanisms driving or impeding the aging process.
